# Interdisciplinary all-on-four® concept for mandibular jaw in dental education - do students benefit from individual 3d printed models from real patient cases?

**DOI:** 10.1186/s40729-024-00528-z

**Published:** 2024-03-12

**Authors:** Monika Bjelopavlovic, Elisabeth Goetze, Peer W Kämmerer, Herbert Scheller

**Affiliations:** 1 Department of Prosthetic Dentistry, University Medical Center of the Johannes Gutenberg-University Mainz, Augustusplatz 2, 55131 Mainz, Germany; 2 Clinic of Cranio-Maxillofacial and Oral Surgery, University Hospital Zurich, University of Zurich, Rämistrasse 100, Zürich, 8091 Switzerland; 3 Department of Oral and Maxillofacial Surgery, University Medical Center of the Johannes-Gutenberg University, Augustusplatz 2, 55131 Mainz, Germany

**Keywords:** 3-D printing, Dental implantation, Dental students, Questionnaires, Dental education, Curriculum development, Hands-on course

## Abstract

**Purpose:**

Digitalization is assuming increasing significance in dental education, as dental students are increasingly exposed to digital implant planning and contemporary technologies such as 3D printing. In this study, we present a cohort analysis aimed at assessing the potential benefits derived from the utilization of 3D prints to seamlessly translate planned procedures into real-life applications.

**Methods:**

21 dental students participated in a virtual planning and hands-on course across two cohorts (C1: *n* = 10, C2: *n* = 11). The virtual implant planning phase involved the placement of four implants on an atrophic lower jaw model. Subsequently, Cohort 1 (C1) executed the implantation procedure on a prefabricated hands-on model, while Cohort 2 (C2) engaged with 3D prints representing their individual implant planning during the hands-on session. Subjective assessments of knowledge, skills, and the perceived utility of 3D prints were conducted through pre- and post-course questionnaires, utilizing a 5-point scale.

**Results:**

In the subjective evaluation, 17 out of 21 participants expressed a positive appraisal of the use of personalized models. Notably, there was no statistically significant improvement in overall knowledge scores; however, there was a discernible increase of 0.5 points in the ratings related to perceived expertise and procedural abilities.

**Conclusion:**

While there was a notable increase in the subjective ratings of knowledge and abilities, no statistically significant difference was observed between the two groups. The consensus among dental students is that individually planned and printed implant models serve as a valuable and effective tool in hands-on courses.

**Supplementary Information:**

The online version contains supplementary material available at 10.1186/s40729-024-00528-z.

## Background

Dental education has undergone significant transformations over the years, with a notable expansion beyond fundamental prosthetic and conservative dentistry topics to incorporate essential elements of implantology and digital procedures. The ongoing trend towards virtual planning and the integration of additive manufacturing to execute treatment plans for patients necessitates adaptation within contemporary dental education. Consequently, teaching staff are increasingly confronted with the imperative to embrace digital learning and incorporate new technologies, ensuring that dental education remains abreast of the latest advancements in the field.

Throughout the years, implantology has become one of the key concepts used for the dental rehabilitation of edentulous patients. Early integration is recommended to effectively complement traditional prosthetic procedures, highlighting the evolving importance of implantology in the comprehensive education of dental students.

Traditional implant concepts, categorized as forward- and backward-planning [1, 2], involve either implant placement focused on the available bone, irrespective of the subsequent prosthetic plan, or implant planning and placement aligned with the prosthetic plan for eventual prosthetic restoration. The latter approach can be implemented through conventional casts and guides manufactured in the laboratory based on patient impressions. Alternatively, virtual planning can be conducted utilizing a three-dimensional x-ray dataset and, if available, 3D-scans of the intended prosthetics or virtually created teeth [3, 4].

It is acknowledged that 3D-planned surgical guides contribute to enhanced precision in surgical implant placement, irrespective of the user’s experience level [5, 6]. Precise preliminary planning, which includes a template for implantation, holds particular significance for complex cases such as so-called all-on-four® restorations [7, 8]. The restoration of edentulous jaws using dental implants, notably through the full arch concept, signifies a substantial progression in contemporary dental practice [9]. This treatment approach involves the strategic placement of four implants in specific locations within the atrophic jaw, allowing for the support of a full-arch fixed prosthesis. By distributing load-bearing forces effectively, the all-on-four® technique provides stability and functionality, enhancing patient comfort and satisfaction [10, 11]. This treatment modality not only offers a reliable solution for edentulous patients but also minimizes the need for extensive bone grafting procedures, thereby reducing treatment complexity, and improving overall treatment outcomes. Consequently, teaching these potential concepts to an ageing patient population is important in the clinical training of students [12, 13]. Within this treatment, 3D printing presents a viable option for generating surgical guides directly within the dental office, with comparable precision to subtractive milled guides or other commercially available solutions [14]. The inherent advantages of in-office 3D printing, including significantly reduced costs, render 3D-printed models and guides highly valuable assets in the field of implant dentistry [15, 16]. 3D printing has furthermore demonstrated its efficacy as a valuable asset in both dental and medical education. Its utilization can significantly enhance comprehension of intricate anatomical structures and streamline communication and training interactions between educators and students, as highlighted by Oberoi [17]. Notably, 3D printing has been widely accepted and positively evaluated by dental students [18–20]. These prints serve as tactile models for simulation, ensuring an authentic haptic experience, while also offering a cost-effective means to produce surgical guides [21].

The integration of traditional implant planning into dental education curricula lacks uniformity, and although initial strides have been made [22], there exists no standardized curriculum for the incorporation of dental implant planning and CAD/CAM-guided implantology utilizing 3D prints [23, 24]. According to the European consensus established by the “Association of Dental Education in Europe,” proficiency in handling pertinent digital data in implantology, including radiology, surgical guides, and diagnostic wax-ups, is identified as a competency goal that should be incorporated into dental education [25]. Notably, the consensus does not explicitly mention familiarity with specific CAD/CAM procedures or virtual planning. The significance of this recommendation is underscored by the observation that, five years after the consensus was formulated, digital procedures and hands-on courses in implantology were infrequently integrated into routine dental curricula [26]. In contrast, the Japanese dental education guidelines advocate for computer-based surgical dentistry as an essential component of dental school education [27], mandating the inclusion of CAD/CAM procedures in dental curricula. This discrepancy highlights the varying emphasis placed on digital technologies in dental education across different regions, underscoring the need for further evaluation and potential standardization to ensure that dental students receive comprehensive training in contemporary techniques and technologies.

In the current landscape of dental education, a survey conducted in Germany in 2011 by the AKWLZ (Arbeitskreis für die Weiterentwicklung in der zahnmedizinischen Lehre = Working group for the further development of teaching in dentistry) and VHZMK (Vereinigung der Hochschullehrer für Zahn-, Mund- und Kieferheilkunde = Association of University Teachers of Dentistry, Oral and Maxillofacial Medicine) revealed that only one CAD/CAM course is regularly integrated into dental university teaching [28]. Conversely, a Canadian survey from 2016 indicated that digital planning and drilling guides are widely implemented across dental education institutions nationwide [29]. Additionally, Koole et al. demonstrated a highly variable approach to didactics in implantology within the United States, with theoretical knowledge being predominantly conveyed, occasionally supplemented by preclinical hands-on experiences, and clinical applications being the least frequently encountered [30]. The topics of CAD/CAM procedures and digitalization were not explicitly analyzed, but the author deemed them highly relevant and suggested further investigation. This observation underscores the prevailing gap between the recommended integration of digitalization and CAD/CAM procedures in dental implantology education and the limited availability of courses addressing these aspects. Furthermore in a German survey study from 2020 shows that undergraduate implantology training as part of dental teaching is perceived very positively by students and postgraduates, but is viewed critically by teachers and practicing dentists [31]. When practical courses are utilized, students report enhanced comprehension, heightened self-awareness, and increased satisfaction [32–34]. Students who have undergone practical training exhibit greater confidence and receptiveness towards the utilization of implantology [22]. The motivation and acceptance levels of students significantly impact the success of their learning endeavors. Moreover, translating theoretical concepts into practical experiences facilitates deeper learning by engaging higher cognitive faculties [35, 36]. While the Likert Scale is commonly used to assess such outcomes [37], its suitability may be questioned. Nevertheless, its widespread usage and simplicity make it a popular choice.

The imperative to bolster implantology education in dental schools and equip dental students with CAD/CAM procedures, including the utilization of 3D-printed devices, warrants further investigation. To address this need, we conducted a pilot study aimed at evaluating whether dental students benefit, in terms of acceptance and subjective knowledge acquisition, from virtual planning of dental implants supplemented by fully customized 3D-printed devices in a hands-on course setting using real case patients.

## Materials and methods

### Study Group

In an exploratory prospective cohort study, a questionnaire evaluation was conducted with dental students participating in an implant course. The study was conducted within the framework of regular dental training at the university medical center, specifically in the department of prosthetics, in collaboration between prosthetics and oromaxillofacial surgery.

Over two consecutive semester terms, students in the regular prosthetic course of the 10th grade (9th semester) were given the opportunity to partake in an implant course. The first semester of participation was designated as cohort 1 (C1, control group), while the subsequent semester involved students participating in the study, designated as cohort 2 (C2, intervention group).

### Teaching concept

**Fig. 1:**
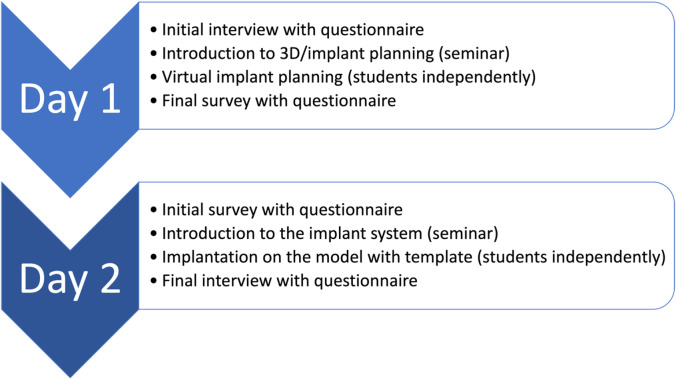
Flowchart of the course timeline

The course was conducted over two separate days (see Fig. 1). On the first day, students attended theoretical lectures on virtual planning in dentistry, received an introduction to the software program used, and engaged in virtual planning using the Nobel Clinician® system (Nobel Biocare AG, Zürich, Switzerland). Patient data, including cone beam CT (CBCT) scans of the edentulous lower jaw and 3D surface scans of the denture, were obtained from the department’s patient pool and anonymized before being provided to the students. The students were instructed to perform implant planning following the all-on-four® concept, utilizing four implants with laterally angulated positions to fully support a fixed lower jaw denture [38]. On the second day, students received a brief introduction to the guided implant system (Nobel Guide®, Nobel Biocare AG, Zürich, Switzerland) and participated in a hands-on course for implant placement using four implants. In total, all students worked on the same patient case and no dentures were made.

**Fig. 2:**
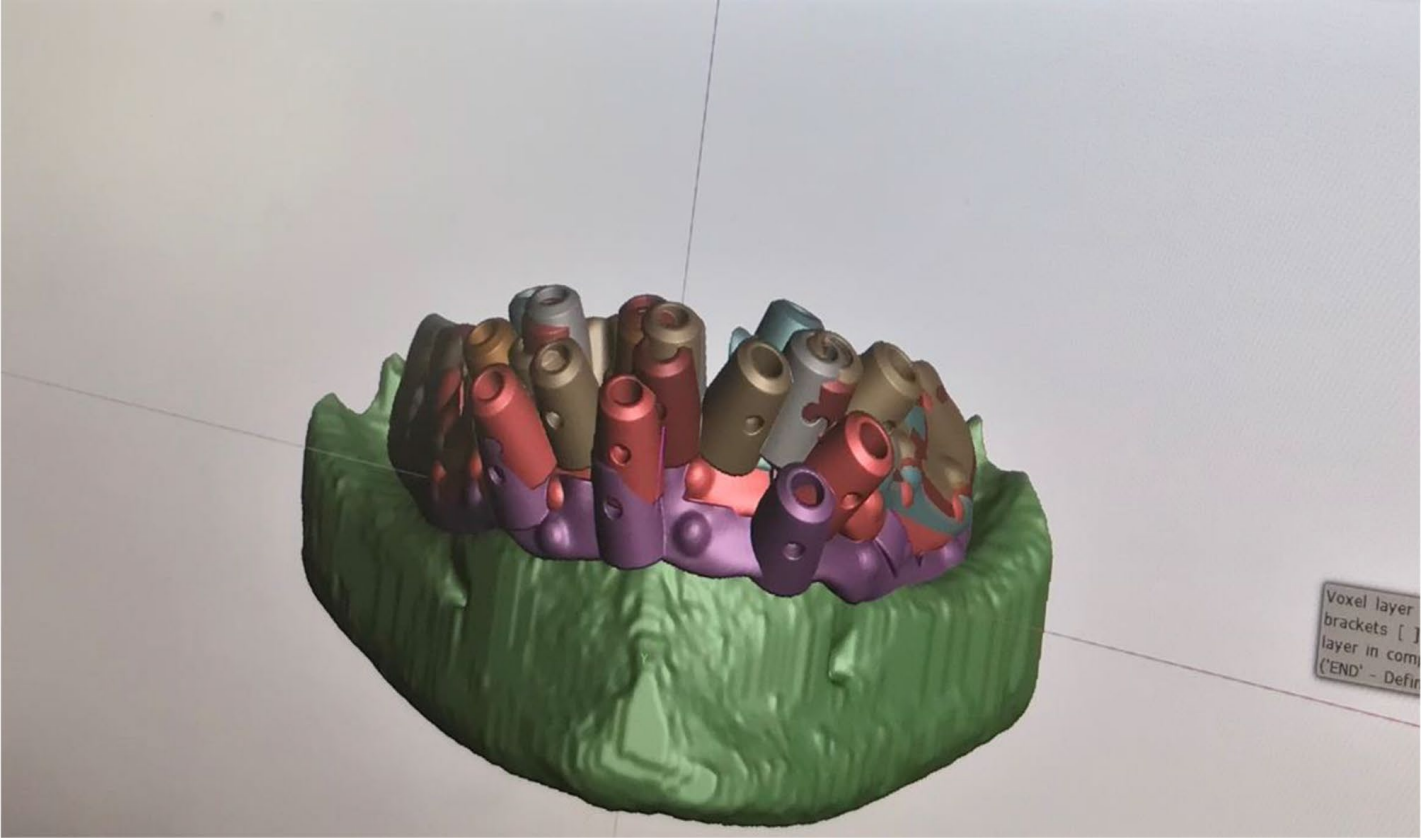
Virtually planned implants of the students with individual templates on the lower jaw of the same patient case

**Fig. 3:**
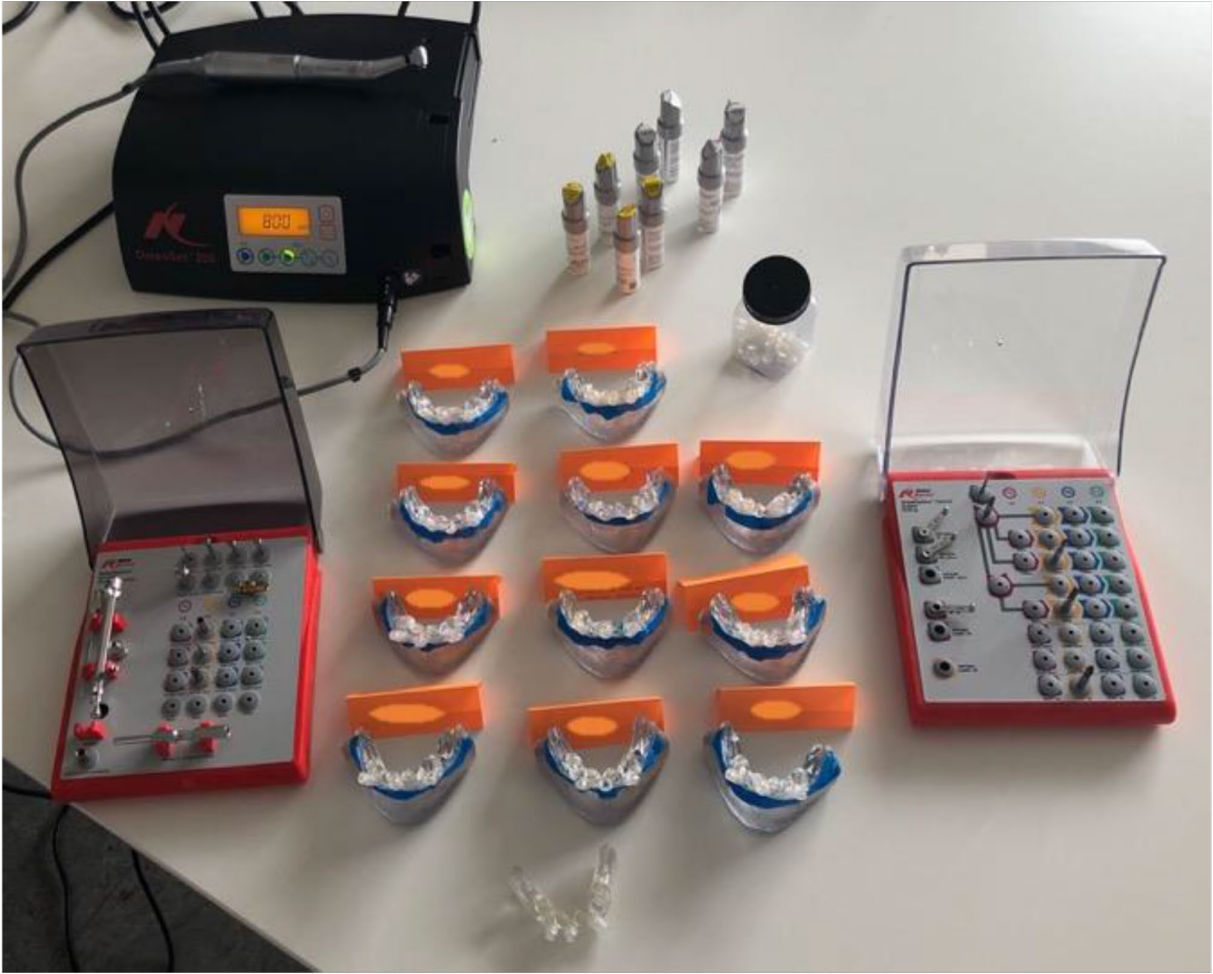
Overview of course materials for cohort 2 included individualized models, the necessary machinery, an implantation set, dummy implants, models equipped with a gingiva mask, and drilling guides. To ensure confidentiality and data protection, the students’ names were anonymized.Overview of course materials for cohort 2 (individualized models, machine, implantation set, dummy implants, models with gingiva mask and drilling guides); the students names are blinded due to data-protection

For the intervention group, the planned implant guides from the first day were exported and 3D-printed, along with the lower jaw, using a polymer printer (Stratasys, Eden 260 V, Eden Prairie, Minnesota, US, Material: MED 610®) (Fig. 2). In contrast, the control group utilized prefabricated jaw models and implant guides for the all-on-four® setup. Both the prefabricated models and the 3D-printed models were equipped with a gingiva mask. Both groups utilized the Nobel Guide® system with Nobel Active® dummy implants for implantation following the standard drilling protocol for guided Nobel Active® implants (see Fig. 3).

### Questionnaire

A questionnaire was administered both before and after the course days to assess students’ acceptance and knowledge gain. Participation was voluntary, and by completing the questionnaire, students consented to participate in the study. However, course participation was possible without completing the questionnaire.

The study group developed the questionnaire in advance and validated it through a trial run during a previous semester’s student course, with input from dental instructors not involved in the study. Any potential issues, such as vague or imprecise questions, were identified and adjusted accordingly.

Data collection was anonymized. The questionnaire included inquiries into basic demographics (such as age, gender, and implant experience). Self-assessment of knowledge related to 3D planning, CAD/CAM procedures, and implant placement was evaluated before and after the course days using a Likert scale [39] (a 5-point rating scale: 1 = very good, 2 = good, 3 = mediocre, 4 = quite bad, 5 = really bad).

Furthermore, following the first course day, students were assessed for knowledge gain and subjective value regarding self-performed virtual planning using rating scales. After the second course day, students were asked to rate the subjective value of individually printed models based on their own planning using a rating scale.

### Statistical analysis

Statistical analysis was done descriptively for demographics and univariate using mean (m), median (md), standard deviation (sd), minimum (min), maximum (max), and quartiles (q). Exploratory data analysis was performed using a Student’s t-test, with a significance level of *p* < 0.05 considered strong evidence for rejecting the null hypothesis (H0). Given the pilot nature of the study, sample size calculation was not feasible beforehand. Therefore, a post-hoc power analysis was conducted afterward, considering alpha values of 0.1 and 0.05, along with power levels of 70%, 80%, and 90%.

## Results

A total of 21 participants were enrolled in the study, with cohort 1 (C1) comprising 11 participants and cohort 2 (C2) comprising 10 participants. The average age of the participants was 27 ± 4 years, with C1 slightly younger at 26 ± 3 years compared to C2 at 28 ± 5 years. The gender distribution was 1:3 (male: female). Responder analysis for the student course, involving 24 students (12 per semester), revealed response rates of 83% and 92%, respectively. However, a non-responder analysis was not conducted. Among the participants, only one individual had a primary medical/dental education. Approximately 57% (12/21) had received prior implant-associated instruction through hands-on courses, while 52% (11/21) had previously assisted in surgical dental implant placements. Moreover, 65% of the students had received some form of training in implantology. Refer to Table 1 for a summary of these demographics.

**Table 1:** Summary of preliminary education and implantology experience

Item	Overall *n* = 21	Control group *n* = 10	Intervention group *n* = 11
Preceding education	*n* = 1 (medical doctor) missing: none	none	*n* = 1
Previous implantation on model	Yes: 12 (57,1%) No: 7 (33,3%) Missing: 2	Yes: 6 (30%) No: 3 (60%) Missing: 1	Yes: 6 (54,6%) No: 4 (36,4%) Missing: 1
Previous Implantation assisted	Yes: 11 (52%) No: 9 (43%) Missing: 1	Yes: 7 (70%) No: 3 (30%) Missing: 0	Yes: 4 (36,4%) No: 6 (54,6%) Missing: 1
Previous implantation	Yes: 1 (4,8%) No: 19 (90,5%) Missing: 1	Yes: 0 (0%) No: 10 (100%) Missing: none	Yes: 1 (9,1%) No: 9 (81,8%) Missing: 1

**Fig. 4:**
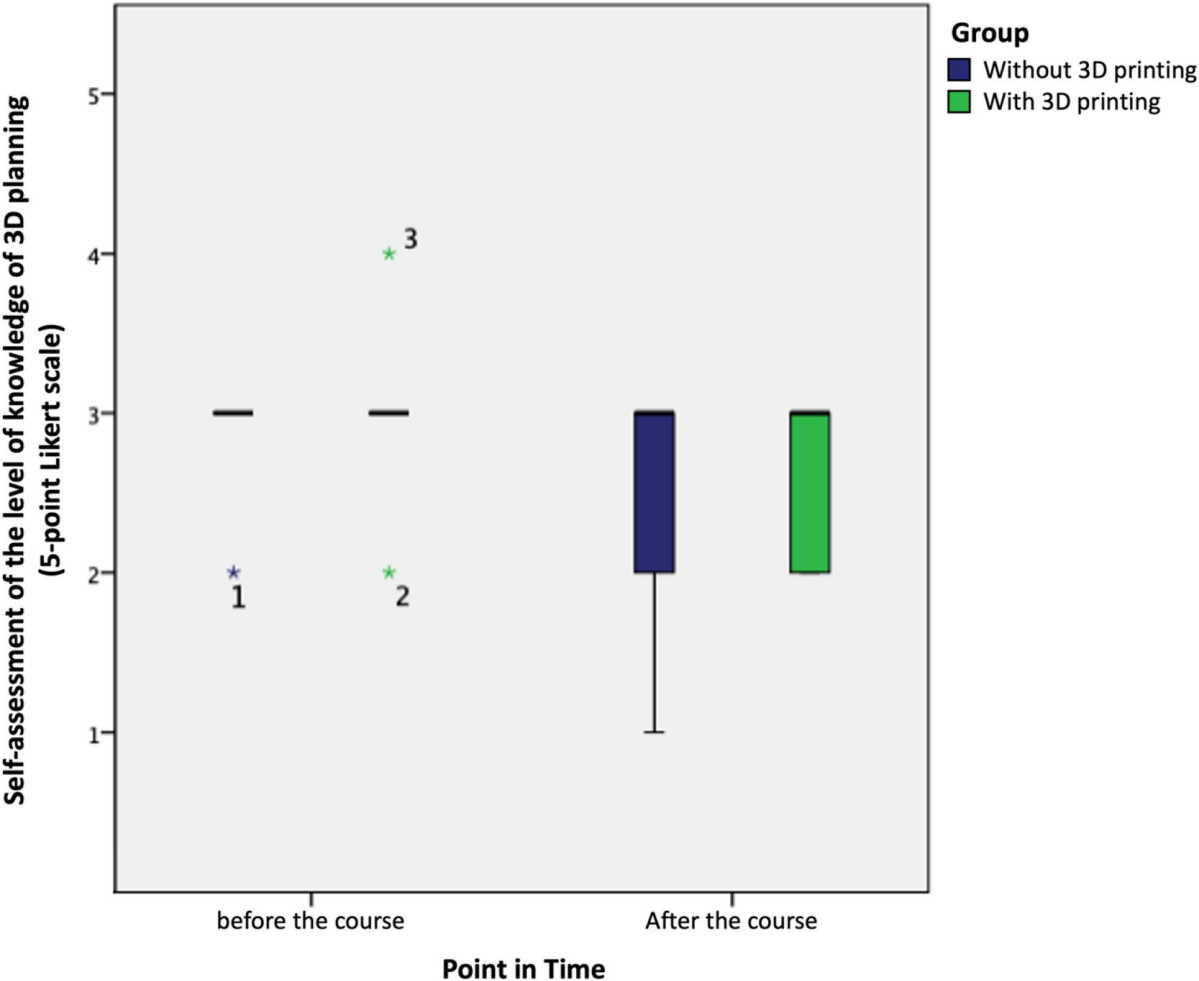
Boxplot for self-assessment of knowledge regarding 3D-planning compared for the control (no 3D-print, *n* = 10) and intervention group (using 3D-print, *n* = 11) separated for evaluation point (before and after the course)

**Fig. 5:**
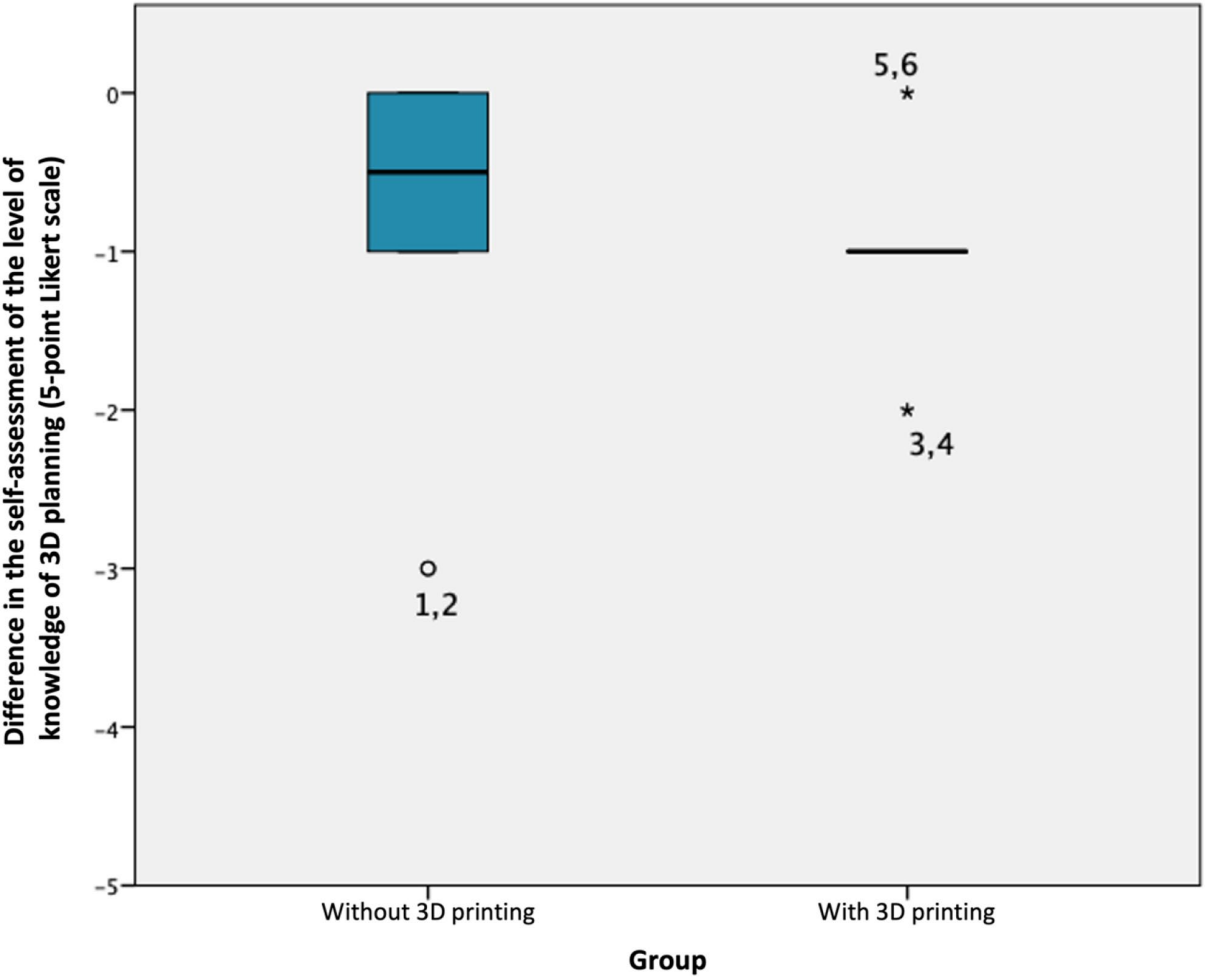
Difference-boxplot for self-assessment of knowledge of 3D-planning compared for the control (no 3D-print, *n* = 10) and intervention group (using 3D-print, *n* = 11)

The subjective assessment of knowledge in 3D planning, measured using a Likert scale ranging from 1 (very good) to 5 (really bad), decreased from an average score of 3.0 ± 0.4 before the course to 2.9 ± 1.3 after the course for both cohorts (see Fig. 4). When analyzed by subgroups, both cohorts exhibited a median decrease in expertise ranging from − 0.5 to -1 points (C1: -0.5 points, C2: -1 point) without a statistically significant difference between the groups (*p* = 0.64) (see Fig. 5). Furthermore, there was no difference in knowledge level observed between students with or without prior implant training, both overall and when compared between C1 and C2.

**Fig. 6:**
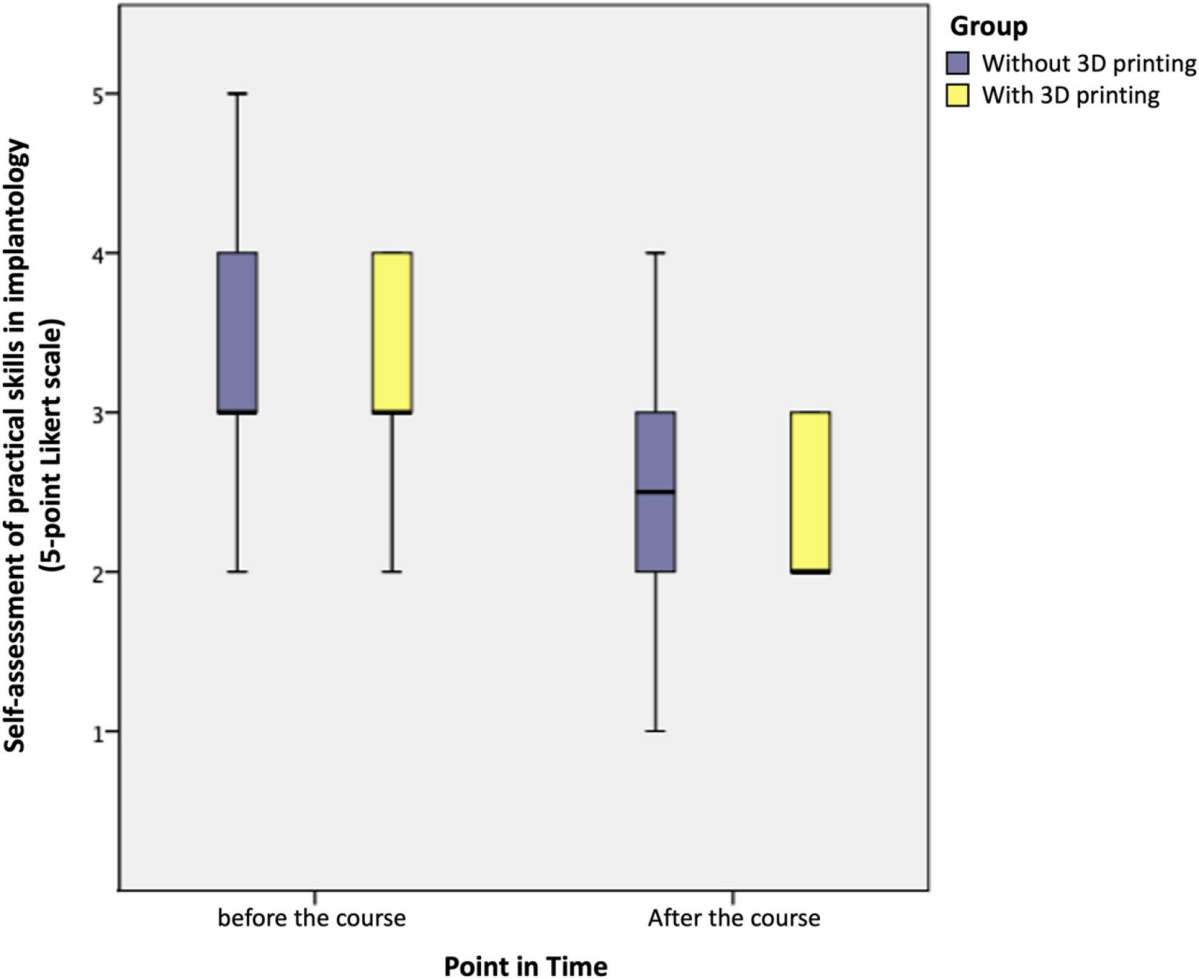
Boxplot for self-assessment of implantology abilities compared for the control (no 3D-print, *n* = 10) and intervention group (using 3D-print, *n* = 11) separated for evaluation point (before and after the course)

The initial knowledge level in implantology was 3.3 ± 0.9, which improved to 2.4 ± 0.9 on average after the course. On average, students gained one rating point in practical skills, with C1 showing an improvement of -0.5 points compared to -1 point for C2 (see Fig. 6).

The majority of students (81%) found the use of individualized models in the course to be valuable, regardless of cohort. Additionally, 90.5% expressed a desire for such a course to be implemented in the regular dental curriculum, with 38% preferring an earlier implementation. Refer to Table 2 for a summary of the course evaluation.

**Table 2:** Summary of course evaluation

Item	Overall *n* = 21	Control group *n* = 10	Intervention group *n* = 11
Individualized model valuable	Yes: 17 (81%) No: 2 (9,5%) Missing: 2	Yes: 8 (80%) No: 2 (20%) Missing: none	Yes: 9 (81,8%) No: 0 (0%) Missing: 2
Desire for implementation of course in regular dental curriculum	Yes: 19 (90,5%) No: none Missing: 2 (9,5%)	Yes: 10 (100%) No: 0 (0%) Missing: none	Yes: 9 (81,8%) No: 0 (0%) Missing: 2
Desire for earlier course integration	Yes: 8 (38,1%) No: 11 (52,4%) Missing: 2 (9,5%)	Yes: 4 (40%) No: 6 (60%) Missing: 0	Yes: 4 (36,7%) No: 5 (45,5%) Missing: 2

A post-hoc analysis was conducted to assess the effect of the low number of participants. Estimating an alpha of 0.05, a power of 80%, and a detectable measurement of 0.5 rating points, a sample size of *n* = 34 was determined to be necessary.

## Discussion

Although the analysis does not corroborate the primary hypothesis suggesting greater subjective knowledge gain among students from an individually planned and model-transferred hands-on course compared to a traditional model course, the present study elucidates several significant observations.

Notably, students’ acceptance of an individualized course is notably high, as evidenced by an 81% acceptance rate in the current study. This finding underscores the potential benefits of implementing such courses, as heightened acceptance among students is recognized to enhance learning capabilities [40]. Despite the absence of statistical significance in the data regarding the usefulness of an individually planned jaw model for implant-course use, students consistently rated the individualized model as helpful and desirable. This subjective perception among students may significantly enhance motivation in learning digital implantology, thereby facilitating the acquisition of application-specific knowledge [22, 41].

Implantology has become an indispensable component of contemporary dental practice, with its principles firmly entrenched in the national learning curriculum for dentists. However, practical implant courses are not universally emphasized. Students appear to recognize this gap, as evidenced by a high proportion (65%) within the study population reporting personal involvement in either planning or assisting with implant procedures. Consequently, it appears that the demand for implantological education remains unfulfilled within dental education programs. A 2021 study showed that students who were allowed to implant under supervision had comparable results to postgraduate dentists [42]. These results are also supported by a study which showed an implant survival rate of 98% for 294 implants placed by students in the University of Kentucky [43]. In both studies, various patient scenarios were employed for training purposes. These studies explored the implantation of four support implants in edentulous patients to facilitate a removable prosthetic denture, as well as single-tooth restorations. Similarly, our study examined the restoration of edentulous patients among students through the implementation of the all-on-four concept®, albeit without subsequent fixed denture—a procedure of highest complexity. Nonetheless, this approach encompasses all essential tools, both theoretically and practically, necessary for comprehensive implantation planning, thus ensuring a proficient execution of the procedure. Studies have indicated that students express a desire for implantology-focused education during their undergraduate dental studies, a sentiment that was positively evaluated by Schweyen et al. [31]. However, the integration of such programs is not constantly detectable. This phenomenon may be attributed in part to economic considerations. Hands-on implant courses are seldom organized without the backing of industrial partners. The expenses associated with machines, burs, dental implants or implant dummies, and jaw models must be taken into account when devising such a course model, particularly given that implants and models are typically single-use items. Many departments lack the resources necessary for the regular implementation of hands-on courses independently [26, 27]. Utilizing low-budget 3D printers presents an opportunity to mitigate costs, as models can be produced more affordably compared to commercially available alternatives [2, 44].

A rough estimation for the current study revealed that costs were approximately €20 per model, inclusive of the gingiva mask, based solely on material expenses. These costs may ostensibly be comparable to a confectioned jaw model but personnel costs and expenses associated with maintaining a printer system were not factored into this calculation. While these additional costs may slightly increase the overall expenditure, leveraging low-budget 3D printers has the potential to significantly reduce the economic burden of hands-on courses, particularly if printing facilities are already available onsite. Consequently, implementing a simulation course may become a more appealing option for dental schools and university departments.

Simulation courses offer a valuable assessment platform for evaluating student proficiency. Moreover, these methods have been shown to enhance student performance and are effective for teaching procedural skills [8]. Additionally, they provide a risk-free environment for students to practice patient-centered applications.

Simulations in medical education must adhere to specific criteria [8], including:

1. Repeated application feasibility.
2. Potential for curricular integration.
3. Adjustability of difficulty levels.
4. Mappable clinical variation.
5. Controlled practice environment.
6. Individualized, active learning.
7. Adaptability to diverse learning strategies.
8. Availability of tangible and measurable results.
9. Internal feedback mechanism for the course.
10. Validity of the simulation as a surrogate for clinical practice.

For hands-on implant courses, it has been demonstrated that most of these criteria (2, 5, and 7–10) are met [25]. Through the utilization of 3D printed models, it becomes feasible to cost-effectively integrate repeated applications with adjustable difficulty levels, based on real patient cases, into an implant course. This approach facilitates the mapping of clinical variation and allows for individualized learning experiences. From a simulation-didactic standpoint, it appears that a simulation involving self-planned 3D print models would be a valuable addition to dental teaching implant courses.

In the current study, a formative evaluation was not conducted; however, other research teams have demonstrated that similar courses are well-suited for this purpose [27, 37]. Therefore, in future applications, a similar course could potentially be utilized to assess implantological skills and knowledge acquisition, thus warranting consideration for implementation in curricular teaching. Despite the lack of a statistically significant increase in knowledge, the positive perception reported by the students supports this assumption. Nevertheless, before full implementation, it is advisable to reassess the suitability of a course individualized by 3D printing for formative testing. A potential limitation of our study is that students in both cohorts may have exhibited a general satisfaction with the opportunity to participate in an implant course, thereby potentially masking any discernible differences between confectioned jaw models and individual 3D-printed cases. It is conceivable that implementing the implant course earlier in the curriculum, subsequent to students acquiring foundational knowledge with confectioned jaw models, could provide a valuable progression. This approach would afford students the opportunity to practice on their own patients using models, thereby gaining insights into the challenges and constraints inherent in real-world dental practice.

## Conclusion

As digitalization continues to play an increasingly significant role in contemporary dental education, the present study sought to investigate whether students subjectively benefit from the translation of self-planned implantation into an individualized 3D-printed model for hands-on courses.

Although the results did not reveal a statistically significant difference between confectioned models and individualized 3D prints in terms of subjective knowledge gain and abilities, students universally perceived individualized planning and hands-on courses as valuable tools in dental education.

The significance of individualized learning objects in hands-on courses is highly appreciated by students, despite the fact that the subjective assessment of knowledge gain or abilities does not appear to increase based on the utilization of such appliances. Further quantitative evaluation may offer a more precise assessment of the benefits of individualized models, such as drilling precision or time-on-task. Consequently, additional research is warranted to address this question comprehensively.

### Electronic supplementary material

Below is the link to the electronic supplementary material.

Supplementary Material 1

## Data Availability

The data from this study were part of the dissertation paper from E.G. Data can be seen in Figs. 1, 2, 3, 4, 5 and 6; Table 1, and 2 and Appex 1–2.

## Declarations

### Amendments

Course materials (laptops, software, drilling machines, burs, dummy implants, confectioned models, and surgical guides) were supplied with the courtesy of Nobel Biocare®. We would like to thank Mrs. Dagmar Wagner for the support.

## Abbreviations

AKWLZ: Arbeitskreis für die Weiterentwicklung in der zahnmedizinischen Lehre
CBCT: Cone Beam Computed Tomography
CAD/ CAM: Computer-aided-design/ Computer-aided-manufacturing
VHZMK: Vereinigung der Hochschullehrer für Zahn-, Mund- und Kieferheilkunde
