# Supplementary material for: Interdisciplinary all-on-four® concept for mandibular jaw in dental education - do students benefit from individual 3d printed models from real patient cases?
Source: Int J Implant Dent. 2024 Mar 12;10:10. doi: 10.1186/s40729-024-00528-z (PMC10933220; doi:10.1186/s40729-024-00528-z)
Supplement: Supplementary file 1 [file 40729_2024_528_MOESM1_ESM.docx]

Questionnaire Impl.-Course, before   Date:_______________

The participation in the survey is voluntary. By completing the questionnaire, the student agrees that the data collected hereby may be analyzed and published as part of an observational study. The data will be collected anonymously. There will be no disclosure of personal data to third parties.

Please assess your practical skills in implantology:

☐ Very good   ☐ Good   ☐ Moderate   ☐ Bad   ☐ Very Bad

To what extent do you feel prepared for the practical implementation on a model through the previous course?

☐ Very good   ☐ Good   ☐ Moderate   ☐ Bad   ☐ Very Bad

To what extent do you feel prepared for the practical implementation of an implantation through the course and exercises?

☐ Very good   ☐ Good   ☐ Moderate   ☐ Bad   ☐ Very Bad

Please assess your level of knowledge regarding 3D planning in oral and maxillofacial surgery (MKG):

☐ Very good   ☐ Good   ☐ Moderate   ☐ Bad   ☐ Very Bad

Have you ever conducted a model implantation before?

☐ Yes   ☐ No

Have you ever assisted in a patient's implantation procedure?

☐ Yes   ☐ No

Have you ever performed a patient's implantation procedure before?

☐ Yes   ☐ No

Personal Information:

Age: _________

Gender: _________

Academic Semester: _________

Previous Professional Training (Dental Assistant, Dental Technician)

☐ Yes   ☐ No

Questionnaire Impl.-Course, after Date:_______________

The participation in the survey is voluntary. By completing the questionnaire, the student agrees that the data collected hereby may be analyzed and published as part of an observational study. The data will be collected anonymously. There will be no disclosure of personal data to third parties.

Please assess your practical skills in implantology:

☐ Very good ☐ Good ☐ Moderate ☐ Bad ☐ Very Bad

To what extent do you feel prepared for the practical implementation of an implantation through the course and exercises?

☐ Very good ☐ Good ☐ Moderate ☐ Bad ☐ Very Bad

Please assess your level of knowledge regarding 3D planning in oral and maxillofacial surgery (MKG):

☐ Very good ☐ Good ☐ Moderate ☐ Bad ☐ Very Bad

In your opinion, would the implantation on a self-planned model (the case from the previous course day) have been advantageous?

☐ Yes ☐ No

If yes, why:

____

____

Would you integrate this course into regular student education?

☐ Yes ☐ No

Should this course take place at an earlier time?

☐ Yes ☐ No

Personal Information:

Age: _________

Gender: _________

Academic Semester: _________

Previous Professional Training (Dental Assistant, Dental Technician)

☐ Yes ☐ No

Suggestions for improvement of the course:____
